# Digital Health Literacy in Pediatric Rheumatic Disease: A Qualitative Study Using Interpretive Description

**DOI:** 10.3390/children13091271

**Published:** 2026-09-18

**Authors:** Craig Eling, Jennifer N. Stinson, Alan M. Rosenberg, Tristan Kerr, Maryam Mehtar, Jasmin Bhawra, Donna Goodridge, Roona Sinha

**Affiliations:** 1 Faculty of Nursing, University of Regina, Saskatoon Campus, 2225 Northridge Drive, Saskatoon, SK S7L 6X6, Canada; 2 Research Institute, The Hospital for Sick Children, 686 Bay Street, Toronto, ON M5G 0A4, Canada; jennifer.stinson@sickkids.ca; 3 Department of Pediatrics, College of Medicine, University of Saskatchewan, c/o Royal University Hospital, 103 Hospital Drive, Saskatoon, SK S7N 0W8, Canada; alan.rosenberg@usask.ca (A.M.R.); tristan.kerr@saskhealthauthority.ca (T.K.); m.mehtar@usask.ca (M.M.); 4 School of Occupational and Public Health, Toronto Metropolitan University, 350 Victoria Street, Toronto, ON M5B 2K3, Canada; jasmin.bhawra@torontomu.ca; 5 Department of Medicine, College of Medicine, University of Saskatchewan, c/o Royal University Hospital, 103 Hospital Drive, Saskatoon, SK S7N 0X8, Canada; donna.goodridge@usask.ca

**Keywords:** digital health, digital health literacy, health information seeking behavior, pediatric rheumatic diseases, adolescents, caregivers, qualitative research

## Abstract

**Highlights:**

**What are the main findings?**

- Online health information (OHI) is used to manage juvenile idiopathic arthritis issues and for reassurance as an alternative to imposing on their medical team.
- Participants had issues finding satisfactory answers through OHI and demonstrated the consumption of misinformation as a result of inadequate appraisal skills and knowledge, which are essential components of digital health literacy.

**What are the implications of the main findings?**

- Pediatric rheumatology care teams can consider providing OHI prescriptions to improve OHI appraisal skills and provide trustworthy OHI resources for the diverse developmental stages of the pediatric rheumatic disease population.

**Abstract:**

**Background/Objectives**: Online health information (OHI) is used by adolescents and caregivers to manage pediatric rheumatic diseases (PRDs). Little is known about the lived experiences of this group as they navigate complex, evolving online healthcare information and services. This study aimed to explore the lived experiences and indicators of digital health literacy (DHL) among adolescents with PRD and caregivers of children and adolescents with PRD through their use of OHI to learn about and manage their disease. **Methods**: Qualitative interviews using Interpretive Description methodology was utilized. Adolescent and caregiver participants were recruited using purposeful sampling from one rheumatology clinic in Saskatchewan, Canada. After completing a pre-interview questionnaire, one-on-one semi-structured telephone interviews were completed, audio-recorded, transcribed verbatim, and analyzed using constant comparative analysis. **Results**: A total of 10 caregivers and 7 adolescents completed the interviews. Three key themes emerged: (1) OHI offers knowledge without imposing on PRD practitioners. Participants were motivated to use OHI for symptom management, reassurance, and medication administration. (2) Participants had difficulty finding OHI, resulting from their search strategies, desire for answers to complex problems, lack of information for novel biologic medications, the absence of adolescent-focused OHI, and confusion around pediatric to adult disease names. (3) Although aware of misinformation, participants discussed and demonstrated inadequate appraisal of OHI. **Conclusions**: Participants relied on inappropriate indicators of medical accuracy and misplaced trust in OHI sources, leading to exposure to misinformation. Proactively addressing these concerns, including OHI prescriptions, fostering independent OHI appraisal, and development of age-appropriate OHI, may improve DHL and aid PRD management.

## 1. Introduction

In an increasingly digitized healthcare landscape [1], individuals can use technology, digital services, and the internet for health-related education, management, and communication. This is known as digital health, which can provide equitable, accessible, and universal care to meet individual needs [1]. Digital health has the potential to improve health throughout the lifespan and includes multiple facets of health promotion and disease prevention, primary care, rehabilitation, and end-of-life care [1]. Online health information (OHI), a component of digital health, is any health-related information found online. It can be presented simply, such as a text-only website, or be interactive, using multiple media formats and technologies. To gain benefit from OHI, as with any aspect of digital health, users must possess an adequate level of digital health literacy (DHL), which has emerged as a critical determinant of health outcomes [1,2,3]. DHL is the skills and knowledge necessary to access, appraise, understand, and integrate digital information and services to benefit health [2,3]. The specific DHL-related skills and knowledge are acquired throughout a lifetime and evolve depending on age and development stage, motivational factors, and digital health opportunities [4].

Pediatric rheumatic disease (PRD) is an umbrella term for a group of chronic complex musculoskeletal and connective tissue diseases, with juvenile idiopathic arthritis (JIA), systemic lupus erythematosus (lupus), and vasculitis being prominent examples. PRDs can adversely affect various facets of a child’s life, such as growth and development, quality of life, social engagement, and academic performance [3]. High-quality OHI can serve as an adjunct to PRD care, empowering and supporting patients and families to advance PRD knowledge, improve coping strategies, prepare for upcoming clinic visits, and foster independent disease management [3,5].

A recent systematic review of the digital health tools available for juvenile idiopathic arthritis (JIA; the most common subtype of PRD) management by Anton et al. [6] recognized that the target of digital health intervention is the patient and their caregivers, for which qualitative research is the best means to capture their insights, perspectives, emotions, and experiences. This current study aimed to address this knowledge gap by using qualitative interviews to explore the lived experiences of adolescents with PRD and the caregivers of children and adolescents with PRD using OHI to learn about and manage their disease. These two populations will hereon be referred to as families with PRD.

## 2. Materials and Methods

### 2.1. Study Design and Ethical Approval

We utilized Interpretive Description, a methodology that recognizes the training, perspective, and experience of healthcare professionals, to translate participants’ subjective experiences to clinically relevant findings that can be applied to improve and inform practice [7,8]. Recognizing that socio-cultural and healthcare contexts are unique to this study’s geography, we have endeavored to provide rich descriptions of the study population and setting to aid the process of transferability [9]. Ethical approval was granted from the University of Saskatchewan’s Behavioral Research Ethics Board (BEH 4858) and operational approval was granted from the Saskatchewan Health Authority (OA-UofS-4858).

### 2.2. Sample

We aimed to recruit 10 adolescents (12 to 19 years old) [10] with PRD and 10 caregivers of children or adolescents (0 to 19 years old) with PRD. Participants were recruited through purposeful sampling [11] from the pediatric rheumatology clinic at the Jim Pattison Children’s Hospital in Saskatoon, Saskatchewan, Canada. Adolescents with PRD and the primary caregivers of children or adolescents with PRD who were receiving care as in- or out-patients were considered eligible for inclusion as study participants. The term caregiver was inclusive of any adults who provided the bulk of the daily medical and non-medical care. The target sample size is consistent with the recommendations for Interpretive Description [7,8] and the scope of this study, and practical within the setting of Saskatchewan’s patient population. Participants who resided outside of Saskatchewan, who no longer required treatment or follow-up, were not fluent in conversational English, or who did not have access to a telephone were ineligible for inclusion in the study.

Recruitment and the collection of consent and/or assent was done by a research coordinator affiliated with the pediatric rheumatology research program. Potential participants were approached at the time of presentation for pediatric rheumatology care or through an announcement by a pediatric rheumatologist at annual PRD charity fund raising events. Potential participants were informed that their medical treatment would not be affected by their choice to participate or not, and they would be free to withdraw at any time without any consequences. Participants were offered a $25 CAD incentive for participating and a further $50 CAD for member checking of the themes [12,13].

### 2.3. Data Collection

Participants were asked to complete a pre-interview questionnaire to collect demographic, internet use, and PRD disease status data through RedCap electronic data capture tools hosted at the University of Saskatchewan [14,15] (see Files S1 and S2 in the Supplementary Materials for the pre-interview questionnaires for each group). Telephone-based support was offered to all participants. Participants completed semi-structured telephone-based qualitative one-on-one interviews with a researcher independent from the clinical pediatric rheumatology practice at the recruitment hospital (CE). Interviews were audio recorded (see Files S3 and S4 in the Supplementary Materials for the interview guides). At least one adolescent had another person present during their interview, which was only realized through whispered comments on the interview recording when confirming anomalies in that section of the transcript. Interviews occurred between October 2024 and December 2025, with each interview lasting between 14 and 45 min (averaging 22 min for adolescents and 32 min for caregivers).

### 2.4. Data Analysis

Pre-interview questionnaire data was analyzed in Microsoft Excel for Mac version 16 (Microsoft Corporation, Redmond, WA, USA) for descriptive statistics (central tendency, variability, and frequency distribution). Transcripts were generated through voice-to-text technology using Otter.ai (https://otter.ai/, Otter.ai Inc., Mountain View, CA, USA) or Microsoft Word for Microsoft 365 (Microsoft Corporation). All transcripts were checked for authenticity by a researcher (CE) prior to data analysis [16]. NVivo version 15.3 (Lumivero Inc., Denver, CO, USA) was used as a platform to organize and code the transcripts. The draft coding manual was informed by a rapid review of the literature [2,3,17,18] and updated through open coding in an iterative manner as the study progressed [19].

Open coding was used to build codes from fragments of transcripts through an inductive, bottom-up approach using constant comparative analysis, beginning after completion of the first interview [7,8,20]. As the coding progressed to thematic generation, isolated themes were abandoned and the mature themes were member checked by four participants from that same age group prior to finalization [7,8,21]. The finalized themes were compared between age groups by within-method triangulation [7,22].

### 2.5. Rigor

Epistemological integrity was created through consultation and feedback from the coauthors who are experienced in health sciences qualitative research, through previous qualitative research training, and the detailing of the methodological research decisions [7,8]. Representative credibility was produced through our sampling strategy, prolonged engagement with participants over the 18 months of data collection, and use of field notes [7,19]. Analytic logic was maintained through the audit trail of our techniques, coding, and thematic generation [7,20]. Interpretive authority was constructed through member checking, discussion of emerging and final themes with research team members, and critical reflection of personal and research team positioning within the research [7]. Reflexivity was implemented through self-auditing, positionality, peer debriefing, and reflexive journaling by CE, who conducted the interviews and analysis [7,8].

## 3. Results

Ten caregivers and seven adolescents completed the pre-interview questionnaire and interview. Non-responders included six caregivers and eleven adolescents who consented but did not reply to interview requests. Interview participants’ demographic, health, and technology details are shown in Table 1.

### 3.1. Theme 1: OHI Offers Access to Knowledge Without Imposition to the Pediatric Rheumatology Care Team

OHI was used to find immediate answers to emerging PRD questions and for reassurance after health decision-making done in pediatric rheumatology clinics. OHI is an alternative to contacting the pediatric rheumatology care team, which requires waiting for a response: “it’s not always you can call them and [sound of snapping fingers] get them on the phone” (Caregiver 1). Participants were also concerned with possibly interrupting and inconveniencing pediatric rheumatology care team members for a “quick little question” (Adolescent 5). Recognizing that the team is busy and “sometimes you can’t even reach them” (Adolescent 3), OHI was an alternative: “but if you search on the Internet, you can find your answer as quick as that, as quick as a click of a button” (Adolescent 3).

Adolescent participants expressed that PRD symptoms and fear of needles was the motivation to seek answers via OHI. They sought to find “something that, I guess helped me, like, with, with needles… how to do it myself” (Adolescent 6). Likewise, caregivers participating in health-related decision-making while in PRD clinics caused anxiety, stress, and worry. “I still get nerved up (Caregiver 1),” states a caregiver when reflecting on the decisions made in clinic. Another participant discussed this burden through saying “I think I just want, like, reassurance and confidence in whatever decision I’m making… I just always want to be like, really sure that that’s absolutely the best choice” (Caregiver 4).

### 3.2. Theme 2: Difficulty Finding Answers

Both caregiver and adolescent participants experienced inability to find satisfactory answers to their PRD questions and concerns through OHI. “And maybe that’s just because [the PRD symptoms have] never happened to somebody else before… I don’t know” (Caregiver 4). Participants recognized that their search strategies may be the cause: “I guess Google or Safari not completely understanding what you mean by how you word it” (Adolescent 5). Caregivers expressed concerns with the inability to find pediatric-specific information about medications: “there wasn’t a lot of information on my little four-year-old [child], who now had to start taking [a novel medication], right?” (Caregiver 4). Associated with this was the lack of information about the long-term effects of taking certain classes of PRD medications: “we don’t have that information. We don’t have all the information yet” (Caregiver 5).

Adolescent participants spoke of the lack of OHI specific to their age group and educational needs, as they found the existing OHI either too childish or too complex. Medical terminology was similarly a source of confusion: “yes, very confused. It’s just all the different words that come in randomly. It’s just very confusing to adapt to those” (Adolescent 2). Caregivers also recognized the deficiency of adolescent-centric OHI, with one participant stating “yeah, I think we’re missing her demographic for a lot of health [web]sites” (Caregiver 8). JIA naming conventions presented unique problems for participant comprehension, especially around name changes when transitioning from pediatric to adult rheumatic care.

Uh, well, for teenagers like the, I forget what it’s called, if it’s, I don’t know if it’s idiopathic arthritis or like the idiopathic part is the like, the younger version under 18 [years of age] or something. But I searched up something like that, like the full thing that I have instead of just idiopathic arthritis.

(Adolescent 2)

### 3.3. Theme 3: Misinformation Presents Challenges Appraising OHI for Trustworthiness

Participants expressed awareness of the concept of online health misinformation and that all OHI may not be trustworthy. However, some participants doubted that online health misinformation was a significant concern with PRD OHI: “I don’t, I don’t think there’s much misinformation out there, but I could be wrong about juvenile arthritis…” (Caregiver 5). The existence of misinformation, regardless of its prevalence in PRD OHI, impacted the ability to appraise and trust OHI. “You know, there are certain sites that I find more trustworthy, but at the end of the day, I don’t know that what I’m looking for is trustworthy, and what I’m reading is trustworthy” (Caregiver 8).

Adolescents spoke about the reliance on OHI created by “doctors” or “health professionals” as trustworthy. However, when asked further about what type of health professional would be a trustworthy presenter of OHI, participants were not sure. [Interviewer:] “What kind of professional would you be happy with?” [Adolescent 1:] “Oh, I don’t know. Like, yeah, I don’t really know.” Some adolescent participants used social media, especially TikTok [23], to find most of their PRD-related OHI because the information is created by doctors who “show off their medical license, and they talk about themselves before they share information… so you can trust and know what kind of doctor they are” (Adolescent 3). Although, other participants recognized reliance on this can be misleading, as “they could fake being doctors fake… Fake what they their degrees” (Adolescent 7).

Adolescent and caregiver participants spoke about relying on OHI from websites that displayed the HTTPS padlock icon within the search results or on the OHI-containing website. The absence of the padlock icon indicated that the OHI was “not safe or verified at all, then don’t click on it, because it could be false information” (Adolescent 3). Participants expressed a similar reliance that OHI posted by an account with a blue verified account checkmark icon on social media sites was trustworthy.

When pressed to explain the impact of online health misinformation on their health, participants could not provide specific answers. Most responded hesitantly and with superficial answers, liberally using “I don’t know” or “I guess”. However, participants from both groups discussed checking the information with their pediatric rheumatology care team before assimilating information into their daily health routines.

Perhaps the most poignant statements related to online health misinformation were made by a caregiver participant, who stated with confidence that they have not been affected by misinformation. “Um, no, I don’t think so. Okay, and unless I’ve been misinformed, maybe I felt, maybe I did fall into a trap, and I haven’t” (Caregiver 7). Later in the interview, the participant states that their child with PRD is no longer vaccinated, in response to information found online. “You know, there’s a lot of misinformation that goes around. Or like the vaccine debate… so I don’t vaccinate anymore” (Caregiver 7).

## 4. Discussion

To our knowledge, this is the first study using qualitative methodology to investigate DHL and OHI use in the PRD population. Using Interpretive Description, we were able to gain valuable insights that address our research question, despite the smaller sample size [7,8]. OHI is the preferred source of health information because of the anonymity, convenience, and immediacy of finding information for children and adolescents with chronic diseases and their caregivers [4,18,24,25,26]. Emerging disease symptoms and impending treatments, and the need for reassurance after participating in health-related decision-making was found to be motivating factors for OHI use in other studies [27,28,29]. Hesitancy to interrupt or inconvenience medical teams with health questions is a documented phenomenon related to the provider–patient power differential and patients not wanting to be seen as difficult individuals [30]. Likewise, the delay in receiving a response from the team was further motivation to use OHI for the participants in our study, as has been reported for other patient populations [4,18,25].

However, OHI seeking behavior presents further issues. Ineffective search strategies that fail to locate useful OHI is a symptom of lower levels of DHL [17,18]. Furthermore, considering that education and comprehension levels vary with developmental changes during adolescent years, it is possible participants encountered and dismissed adolescent-specific OHI that did not meet their precise needs [4,24]. This presents as a significant challenge to provide beneficial OHI for the needs of all adolescents. When unable to find OHI that met their needs, adolescent participants admitted to struggling with the complexity of adult-focused OHI [4,24,25,31]. The availability of adolescent-specific OHI has been found to increase empowerment, promote independence and self-efficiency, improve DHL, provide the anonymity to seek answers for sensitive health questions, address unmet needs for health information, enable active participation in health-related decision-making, improve disease management at school, and improve quality of life [4,24,31,32].

PRD is frequently managed with biologic medications, many of which are novel medications. Biologic medications mimic the natural functioning of the body to block the immune response that causes PRD [33]. While these medications have been approved for treating PRD, the nature of novel medications means that abundant real-world data associated with long-term use is not available for the PRD population via OHI. Likely, adolescents and caregivers are expecting to find this information for a specifically prescribed medication, rather than viewing available long-term information about classes of biologic medication, such as pro-inflammatory cytokine inhibitors [33]. This finding suggests that pediatric rheumatology care teams have room for further clarification of long-term safety of biologic medications regardless of individual novel medications prescribed.

Disease names for those diagnosed with JIA was associated with confusion when searching for useful OHI. JIA is divided into seven clinically distinct categories, most of which are re-named after transitioning to adult care [34,35]. These names are often significantly different, such as systemic JIA being renamed to adult-onset Still’s disease, even though the onset was in childhood. However, this practice varies among sites, as a standardized naming convention has not yet been created, despite recognition of the need for one [34].

Participants’ lack of awareness of online health misinformation and the potential impact it has on health was a concerning finding. As appraisal of OHI is a component of DHL, this indicates that participants in our study had inadequate DHL [2,3,17]. The DHL-related skill of appraisal is an active process, involving the application of critical thinking to review the OHI, the author, and mode of presentation to determine reliability [24,29]. Two recent systemic reviews [18,25] found that misinformation was detrimental to the patient–provider relationship, contributed to decreased treatment plan adherence, caused health-related anxiety, and influenced patient use of non-scientifically proven alternative health treatments.

Participants in our study relied on superficial and irrelevant indicators of OHI trustworthiness. Both the HTTPS padlock icon, an indicator of secure online data transmission [36], and the social media blue checkmark icon, indicating a verified account [37], do not have any correlation with the medical accuracy of OHI and often mislead adolescents and caregivers [24,25,31]. A systematic review by Freeman et al. [31] examining how adolescents trust OHI on social media also found reliance on checkmark icon as an indicator of OHI medical accuracy and trustworthiness. Likewise, previously reported studies identified an inappropriate reliance on social media content creators identifying as doctors and other healthcare professionals and the OHI they presented [4,24,26,31,32]. Specifically, in addition to medical doctors, individuals such as dentists, veterinarians, people with a PhD, naturopathic practitioners, and chiropractors can identify as being a doctor [24]. Even though the OHI presented by such individuals may be valid and reliable, they may not be qualified to offer PRD-related medical advice [38]. The complexity of appraising the credentials and licensing of OHI creators is complex, often beyond the understanding of adolescents [24]. Regardless, participants in this study were using an inappropriate reliance on indicators of trustworthiness instead of critically appraising the OHI, indicating a deficit in DHL [24,29].

Most participants in our study desired to discuss OHI, legitimate or otherwise, with the pediatric rheumatology care team prior to acting upon it. Coupled with the willingness of participants to contact the team with questions and the team’s willingness to receive and address such questions, these factors may present as a safety net to online health misinformation for most of our participants, as has been found in other studies [17,18,25,31,32]. Otherwise, the acceptance of online health misinformation can delay symptom resolution, promote anxiety, or cause negative health outcomes [25,31].

### Limitations

The small sample size of both adolescents and caregivers is a possible limitation of this study. The group of participants also had a low amount of ethnic, socioeconomic, and geographical location diversity. As our research team consisted of PRD and non-PRD medical professionals providing care within the same pediatric hospital we recruited in, participants may be biased through social desirability. The higher number of non-responders possibly introduced further bias to this study through a selection bias. These factors limit the generalizability of these findings. We also recognize a power differential exists between the investigators, most of whom are clinicians, including some on the pediatric rheumatology team caring for study participants.

## 5. Conclusions

Families with PRD use OHI to learn about, manage, problem solve, and attain reassurance in their health-related decision-making. The findings from this qualitative interview study indicate that adolescent and caregiver participants have some barriers to their DHL that manifest in their OHI seeking behavior. These include an inability to confidently discern online health misinformation from valid OHI, the reliance on false and superficial indicators of OHI trustworthiness, and a misplaced trust in OHI presented by individuals identifying as a doctor. The creation of OHI that is tailored for adolescents in different stages of development and educational needs would further benefit families with PRD. Whenever possible, we recommend pediatric rheumatology care teams provide DHL-improving OHI prescriptions to families with PRD.

## Figures and Tables

**Table 1:** Demographic, health, and technology details of interview participants, *n* (%, unless otherwise noted).

Demographic Details			
	Caregivers (*n* = 10)	Adolescents (*n* = 7)	
**Age**			
Range	36–48	14–16	
Mean (SD)	42.8 (3.9)	15.7 (1.0)	
**Sex**			
Female	7 (70.0)	4 (57.1)	
**Distance to nearest hospital**			
<50 Km	10 (100)	7 (100)	
**Ethnicity**			
White	9 (90)	6 (85.7)	
Aboriginal	1 (10.0)	0	
Métis	0	1 (14.3)	
**Caregiver highest level of education**			
High school diploma or equivalent	1 (10.0)		
Trade certificate or diploma	3 (30.0)		
Other non-university certificate or diploma	2 (20.0)		
Bachelor’s degree	3 (30.0)		
Master’s degree	1 (10.0)		
**Health Status**			
	**Caregiver’s Own** **(*n* = 10)**	**Caregiver’s Child with PRD** **(*n* = 10)**	**Adolescent’s Own** **(*n* = 7)**
Fair	1 (10.0)	2 (20.0)	1 (14.3)
Good	7 (70.0)	6 (60.0)	3 (42.9)
Excellent	2 (20.0)	2 (20.0)	3 (42.9)
**Pediatric Rheumatic Disease Status**			
	**Caregiver’s Child with PRD** **(*n* = 10)**	**Adolescent’s Own** **(*n* = 7)**	
Inactive disease	2 (20.0)	1 (14.3)	
Minimal disease activity	4 (40.0)	5 (71.4)	
Moderate disease activity	4 (40.0)	1 (14.3)	
High disease activity	0	0	
**Home Internet Access**			
	**Caregivers (*n* = 10)**	**Adolescents (*n* = 7)**	
**Wired Internet Access**			
Home high speed	7 (70.0)	2 (28.6)	
Home normal speed	2 (20.0)	3 (42.9)	
Not specified	0	2 (28.6)	
**Cellular**			
5G cellular	4 (40.0)	3 (42.9)	
LTE or 3G cellular	1 (10.0)	1 (14.3)	
Not sure/Don’t know	1 (10.0)	0	
Not specified	0	2 (28.6)	
**Devices That Can Access the Internet at Home**			
	**Number of Devices**	**Caregivers (*n* = 10)**	**Adolescents (*n* = 7)**
Computers	0	1 (10.0)	0
	1	1 (10.0)	1 (14.3)
	2	4 (40.0)	3 (42.9)
	3	2 (20.0)	0
	4	1 (10.0)	1 (14.3)
	5	1 (10.0)	0
Smartphones	0	1 (10.0)	0
	1	4 (40.0)	0
	2	0	0
	3	1 (10.0)	3 (42.9)
	4	3 (30.0)	2 (28.6)
	5	1 (10.0)	2 (28.6)
Tablets	0	0	2 (28.6)
	1	2 (20.0)	2 (28.6)
	2	5 (50.0)	3 (42.9)
	3	2 (20.0)	0
	Not specified	1 (10.0)	0
**Hours Online Each Day**			
	**Hours per Day**	**Caregivers (*n* = 10)**	**Adolescents (*n* = 7)**
Personal	1 to 2	6 (60.0)	2 (28.6)
	3 to 4	2 (20.0)	3 (42.9)
	5 to 6	1 (10.0)	0
	7 to 8	0	0
	9 to 10	0	2 (28.6)
	Not specified	1 (10.0)	0
Work	1 to 2	3 (30.0)	1 (14.3)
	3 to 4	3 (30.0)	0
	5 to 6	0	0
	7 to 8	1 (10.0)	0
	9 to 10	1 (10.0)	0
	0	1 (10.0)	6 (85.7)
	Not specified	1 (10.0)	0
School	1 to 2	1 (10.0)	3 (42.9)
	3 to 4	0	4 (57.1)
	0	7 (70.0)	0
	Not specified	3 (30.0)	0
**Last Use of Digital Health**			
	**Caregivers (*n* = 10)**	**Adolescents (*n* = 7)**	
Today	1 (10.0)	0	
2 to 7 days ago	3 (30.0)	1 (14.3)	
>7 days & <1 month ago	3 (30.0)	4 (57.1)	
1 to 3 months ago	3 (30.0)	2 (28.6)	

SD: standard deviation.

## Data Availability

The raw data supporting the conclusions of this article will be made available by the authors upon reasonable request due to ethical restrictions.

## Supplementary Materials

The following supporting information can be downloaded at https://www.mdpi.com/article/10.3390/children13091271/s1, File S1: Adolescent questionnaire; File S2: Caregiver questionnaire; File S3: Adolescent Interview guide; File S4: Caregiver Interview guide.

## Abbreviations

The following abbreviations are used in this manuscript:

DHL	Digital health literacy
JIA	Juvenile idiopathic arthritis
OHI	Online health information
PRD	Pediatric rheumatic diseases
