# Supplementary material for: Digital Health Literacy in Pediatric Rheumatic Disease: A Qualitative Study Using Interpretive Description
Source: Children (Basel). 2026 Sep 18;13(9):1271. doi: 10.3390/children13091271 (PMC13605829; doi:10.3390/children13091271)
Supplement: Supplementary file 1 [file children-13-01271-s001.zip › File S1 - Adolescent questionnaire - DHL in PRD.pdf]

Adolescent pre-interview questionnaire

Please complete the survey below.

If you're having any difficulties filling it out, the researcher (Craig Eling) can help you fill it out. This will be done over the phone, before starting the interview. If you'd like help or have any questions,, please send an email to Craig at [redacted]

Demographic Information

What year were you born in?

____

What is your gender?

- ◯ Man/boy
- ◯ Woman/girl
- ◯ Non-binary
- ◯ Not listed
- ◯ Prefer not to answer

What are the first three digits of your postal code?

____

How far away is the nearest hospital in Saskatchewan to where you live?

- ◯ less than 50 Km
- ◯ 51 to 99 Km
- ◯ more than 99 Km

What is your ethnicity? Select all that apply.

- ☐ White
- ☐ South Asian
- ☐ Chinese
- ☐ Black
- ☐ Filipino
- ☐ Arab
- ☐ Latin American
- ☐ Southeast Asian (e.g., Vietnamese, Cambodian, Laotian, Thai)
- ☐ West Asian (e.g., Iranian, Afghan)
- ☐ Korean
- ☐ Japanese
- ☐ First Nations
- ☐ Métis
- ☐ Inuk (Inuit)
- ☐ Other

What is your education level?

- ◯ Currently in grades 6-8
- ◯ Currently in grades 9-12
- ◯ Currently in trade school or college
- ◯ Currently in university
- ◯ Not currently in school/college/university

What is your employment status? Select all that apply.

- ☐ Full-time student
- ☐ Part-time student
- ☐ Full-time employment
- ☐ Part-time employment
- ☐ Not in school and unemployed

Health Information

How is your health?

- ◯ Excellent
- ◯ Good
- ◯ Fair
- ◯ Poor

What is the health status of your rheumatic disease?

- ◯ Non-active
- ◯ Minimal or low disease activity
- ◯ Moderate disease activity
- ◯ Severe disease activity or difficult-to-treat

Do you have any comorbidities (other non-rheumatic diseases)? This can include any other diagnosis or health condition that is not rheumatic.

- ◯ Yes
- ◯ No

Do your biological parents have a rheumatic disease?

- ◯ Yes, both parents
- ◯ Yes, one parent
- ◯ No
- ◯ Not known/I don't know

Digital Access and Internet Usage

What type of Internet access do you have at home? This your personal internet, not through work or school. Select all that apply.

- ☐ High-speed home access
- ☐ Normal-speed home access
- ☐ Satellite access
- ☐ High-speed cellular plan access (5G)
- ☐ Normal-speed cellular plan access (LTE or 3G)
- ☐ No home access
- ☐ I don't know

Do you access the Internet from somewhere outside your home? If so, where do you do this? This includes using cellular data. Select all that apply.

- ☐ Work
- ☐ School
- ☐ Public library
- ☐ Community agency or centre
- ☐ Someone else's home
- ☐ Somewhere else not listed
- ☐ Nowhere else besides home
- ☐ I don't access the Internet

How many computers (desktop and laptop) that can access the Internet do you have access to at home?

(Place a mark on the scale above)

How many smartphones that can access the Internet do you have access to at home?

(Place a mark on the scale above)

How many tablets that can access the Internet do you have access to at home?

(Place a mark on the scale above)

How many personal hours each day do you spend on the Internet? This not time for work or school. It includes any time online, such as social media, playing games, chatting, posting, videoconferencing, and so on.

(Place a mark on the scale above)

How many work hours each day do you spend on the Internet? Don't count the days you are not working.

(Place a mark on the scale above)

How many school hours each day do you spend on the Internet (including for homework)? Don't count the days you're not doing school work.

(Place a mark on the scale above)

When was the last time you used the Internet for your health? This includes looking up lab results/imaging reports, refiling prescriptions, researching health information, looking up side effects, and so on. This includes both rheumatological and non-rheumatological health information.

- ◯ Today
- ◯ In the last 7 days
- ◯ This month
- ◯ One to three months ago
- ◯ Three months to one year ago
- ◯ More than one year ago
- ◯ Never
