# Supplementary material for: Digital Health Literacy in Pediatric Rheumatic Disease: A Qualitative Study Using Interpretive Description
Source: Children (Basel). 2026 Sep 18;13(9):1271. doi: 10.3390/children13091271 (PMC13605829; doi:10.3390/children13091271)
Supplement: Supplementary file 1 [file children-13-01271-s001.zip › File S2 - Caregiver questionnaire - DHL in PRD.pdf]

Caregiver pre-interview questionnaire

Please complete the survey below.

The questions are about yourself and the children with rheumatic disease that you're primary caregiver for. Primary caregiver means you are one of the adults who is most responsible for helping with the child's rheumatic disease. This includes such things as giving medication, taking the child to the doctor, dealing with symptoms, and so forth. You don't need to be the biologic parent to be the primary caregiver.

If you're having any difficulties filling it out, the researcher (Craig Eling) can help you fill it out. This will be done over the phone, before starting the interview. If you'd like help or have any questions,, please send an email to Craig at [redacted].

Demographic Information

What year were you born in? __________________________________

How many children with rheumatic disease do you care for?
- ◯ 1
- ◯ 2
- ◯ 3
- ◯ 4
- ◯ 5
- ◯ 6

What is the year of birth of the child with rheumatic disease? __________________________________

What is the year of birth of the oldest child with rheumatic disease? __________________________________

What is the year of birth of the second oldest child with rheumatic disease? __________________________________

What is the year of birth of the third oldest child with rheumatic disease? __________________________________

What is the year of birth of the fourth oldest child with rheumatic disease? __________________________________

What is the year of birth of the fifth oldest child with rheumatic disease? __________________________________

What is the year of birth of the youngest child with rheumatic disease? __________________________________

What is your gender?
- ◯ Man
- ◯ Woman
- ◯ Non-binary
- ◯ Not listed
- ◯ Prefer not to answer

What are the first three digits of your postal code? __________________________________

How far away is the nearest hospital in Saskatchewan to where you live?
- ◯ less than 50 Km
- ◯ 51 to 99 Km
- ◯ more than 99 Km

What is your ethnicity? Select all that apply.	☐ White<br>☐ South Asian<br>☐ Chinese<br>☐ Black<br>☐ Filipino<br>☐ Arab<br>☐ Latin American<br>☐ Southeast Asian (e.g., Vietnamese, Cambodian, Laotian, Thai)<br>☐ West Asian (e.g., Iranian, Afghan)<br>☐ Korean<br>☐ Japanese<br>☐ First Nations<br>☐ Métis<br>☐ Inuk (Inuit)<br>☐ Other
What is the highest level of education that you have?	○ Did not graduate high school<br>○ High school diploma or equivalent<br>○ Trade certificate or diploma<br>○ Other non-university certificate or diploma<br>○ Bachelor's degree<br>○ Masters' degree<br>○ PhD<br>○ Professional health services doctorate
What is your employment status? Select all that apply.	☐ Full-time employment<br>☐ Part-time employment<br>☐ Unemployed<br>☐ Retired<br>☐ Full- or part-time student
What is your marital/common-law status?	○ Married<br>○ Common-law<br>○ Never married and not living common law<br>○ Separated and not living common law<br>○ Divorced and not living common law<br>○ Widowed and not living common law
What is the total number of children aged 18 or less that you are primary caregiver for?	________________________

Health Information

How is your health?	○ Excellent<br>○ Good<br>○ Fair<br>○ Poor
Do you have a rheumatic disease?	○ Yes<br>○ No<br>○ Unsure
How is the health of the child with rheumatic disease?	○ Excellent<br>○ Good<br>○ Fair<br>○ Poor

What is the health status of the child's rheumatic disease?	◯ Non-active<br>◯ Minimal or low disease activity<br>◯ Moderate disease activity<br>◯ Severe disease activity or difficult-to-treat
Does the child with rheumatic disease have any comorbidities (other non-rheumatic diseases)? This can include any other diagnosis or health condition that is not rheumatic.	◯ Yes<br>◯ No
How is the health of the oldest child with rheumatic disease?	◯ Excellent<br>◯ Good<br>◯ Fair<br>◯ Poor
What is the health status of the oldest child's rheumatic disease?	◯ Non-active<br>◯ Minimal or low disease activity<br>◯ Moderate disease activity<br>◯ Severe disease activity or difficult-to-treat
Does the oldest child with rheumatic disease have any comorbidities (other non-rheumatic diseases)? This can include any other diagnosis or health condition that is not rheumatic.	◯ Yes<br>◯ No
How is the health of the second oldest child with rheumatic disease?	◯ Excellent<br>◯ Good<br>◯ Fair<br>◯ Poor
What is the health status of the second oldest child's rheumatic disease?	◯ Non-active<br>◯ Minimal or low disease activity<br>◯ Moderate disease activity<br>◯ Severe disease activity or difficult-to-treat
Does the second oldest child with rheumatic disease have any comorbidities (other non-rheumatic diseases)?	◯ Yes<br>◯ No
How is the health of the third oldest child with rheumatic disease?	◯ Excellent<br>◯ Good<br>◯ Fair<br>◯ Poor
What is the health status of the third oldest child's rheumatic disease?	◯ Non-active<br>◯ Minimal or low disease activity<br>◯ Moderate disease activity<br>◯ Severe disease activity or difficult-to-treat
Does the third oldest child with rheumatic disease have any comorbidities (other non-rheumatic diseases)?	◯ Yes<br>◯ No
How is the health of the fourth oldest child with rheumatic disease?	◯ Excellent<br>◯ Good<br>◯ Fair<br>◯ Poor

What is the health status of the fourth oldest child's rheumatic disease?	◯ Non-active<br>◯ Minimal or low disease activity<br>◯ Moderate disease activity<br>◯ Severe disease activity or difficult-to-treat
Does the fourth oldest child with rheumatic disease have any comorbidities (other non-rheumatic diseases)?	◯ Yes<br>◯ No
How is the health of the fifth oldest child with rheumatic disease?	◯ Excellent<br>◯ Good<br>◯ Fair<br>◯ Poor
What is the health status of the fifth oldest child's rheumatic disease?	◯ Non-active<br>◯ Minimal or low disease activity<br>◯ Moderate disease activity<br>◯ Severe disease activity or difficult-to-treat
Does the fifth oldest child with rheumatic disease have any comorbidities (other non-rheumatic diseases)?	◯ Yes<br>◯ No
How is the health of the youngest child with rheumatic disease?	◯ Excellent<br>◯ Good<br>◯ Fair<br>◯ Poor
What is the health status of the youngest child's rheumatic disease?	◯ Non-active<br>◯ Minimal or low disease activity<br>◯ Moderate disease activity<br>◯ Severe disease activity or difficult-to-treat
Does the youngest child with rheumatic disease have any comorbidities (other non-rheumatic diseases)?	◯ Yes<br>◯ No

Digital Access and Internet Usage

What type of Internet access do you have at home? This your personal internet, not through work or school. Select all that apply.	☐ High-speed home access<br>☐ Normal-speed home access<br>☐ Satellite access<br>☐ High-speed cellular plan access (5G)<br>☐ Normal-speed cellular plan access (LTE or 3G)<br>☐ No home access<br>☐ I don't know
Do you access the Internet from somewhere outside your home? If so, where do you do this? This includes using cellular data. Select all that apply.	☐ Work<br>☐ School<br>☐ Public library<br>☐ Community agency or centre<br>☐ Someone else's home<br>☐ Somewhere else not listed<br>☐ Nowhere else besides home<br>☐ I don't access the Internet
How many computers (desktop and laptop) do you own that can access the Internet?	*(Place a mark on the scale above)*

How many smartphones do you own that can access the Internet?

(Place a mark on the scale above)

How many tablets do you own that can access the Internet?

(Place a mark on the scale above)

How many personal hours each day do you spend on the Internet? This not time for work or school. It includes any time online, such as social media, playing games, chatting, posting, videoconferencing, and so on.

(Place a mark on the scale above)

How many work hours each day do you spend on the Internet? Don't count the days you are not working.

(Place a mark on the scale above)

How many school hours each day do you spend on the Internet (including for homework)? Don't count the days you're not doing school work.

(Place a mark on the scale above)

When was the last time you used the Internet for your health? This includes looking up lab results/imaging reports, refiling prescriptions, researching health information, looking up side effects, and so on. This includes both rheumatological and non-rheumatological health information.

- ◯ Today
- ◯ In the last 7 days
- ◯ This month
- ◯ One to three months ago
- ◯ Three months to one year ago
- ◯ More than one year ago
- ◯ Never
