# Supplementary material for: Digital Health Literacy in Pediatric Rheumatic Disease: A Qualitative Study Using Interpretive Description
Source: Children (Basel). 2026 Sep 18;13(9):1271. doi: 10.3390/children13091271 (PMC13605829; doi:10.3390/children13091271)
Supplement: Supplementary file 1 [file children-13-01271-s001.zip › File S3 - Adolescent Interview guide - DHL in PRD.pdf]

Interview Guide – Adolescent participants

Part 1: Background Information and Digital Health Engagement

1. Background Internet use

I'm interested in your regular online activities. What do you usually do when you're on the Internet?

- What sites or apps you are most comfortable using and why?
- How about social media – which ones do you normally use?

How much time do you spend online each day?

- How much of this time is for work or school?

2. Current Use of Digital Health Resources and Motivation

What sort of information do you look up online about your health?

- Can you give me examples such as: about the disease, treatments, side effects, or tips for managing your health at school?
- Tell me how you use social media to learn about your health.

[if older than 14-years – the minimum age to have access to MySaskHealthRecord]: Can you tell me why you do or don't have a Saskatchewan MySaskHealthRecord account? This is the account you can use to look up your test results.

How do you find health information online?

- Search words? Search sites? How do you choose which links to click?
- Do you look for info for your age group, adult-focused, or anything?

If no: Probing questions:

- Please tell me if you have any worries about searching for health information online?
- What would make you feel comfortable using the Internet for your health?
- If no, what would need to change before you started looking for health information online?

If sustained no

- Please tell me if you have issues understanding health information (such as big or confusing words) and give me some examples?
- Can you tell me if you have problems using devices or computers and give me some examples?
- Can you tell me about any worries you have with online privacy?
- Can you tell me about any worries you have with online misinformation?
- Can you tell me if you are worried that your health will get worse if you look for information online?

- Is this because you don’t have the Internet at home? Or if it’s because you don’t have a device that can access the Internet?

This is the end of the interview for those who don’t use the Internet for health information and have no plans to start.

Part 2: Barriers to DHL

3. Perceived Challenges

Tell me about the problems you’ve had finding or using health information online.

- Can you tell me if health information you found online made your health worse?
- Tell how looking for health information online in the past has changed how you look now.
- Have you ever felt overwhelmed, frustrated, or confused by health information online? If so, can you tell me why you felt this way?
  - What did you do about this?
  - Have you ever looked for health information from another country or in another language? If so, tell me why, what country, and what language to looked for.
- Have any of your issues been because you need help using a computer or device? If so, can you tell me about it?
- Has getting Internet access at home been an issue for you? If so, can you tell me about it?

4. Privacy

Do you have worries about other people online finding out information about your or your health?

- What do you do about this?

5. Misinformation

When are you worried about misinformation in health information online?

- What does “misinformation” mean to you?
- Tell me about any health information have you ignored because it may be misinformation?
- Has online misinformation changed your how you feel about your health?

Tell me how you decide if information is good or bad?

Have your doctors or nurses ever been worried about the information you found online? Or have the said something that is different than the information you found online?

- Can you give me an example?
- What were the doctors’/nurses’ concerns?

Part 3: Facilitators of DHL

6. Motivation for Digital Health Engagement: Now that we have talked about the bad, I'd like to discuss the good things about looking for health information online.

Why do you want to use the Internet for your health?

- What are hoping to learn?
- How do you hope this will help you?

7. Perceived Benefits

Tell me about the good things that have happened from finding health information online? Can you tell me about this?

- Has the information you've found ever helped you make choice about your health?

What advice would you give to other young people who are seeking health information online?

8. Usability

What can websites or apps do to help you find and learn about your health?

Tell me what helps you use the Internet for your health?

9. Sharing of Information

How have you shared information your find online with others?

- What type of health information was it? Examples: dealing with side effects, improving their quality of life, advice.

Has anyone told you to stop finding or sharing any online information?

10. Improving Supports

If you were going to get some teaching on the using the Internet for health, what would you want to learn?

Has anyone ever helped you find health information online?

- If yes, can you give me an example?
- What were the best and worst things about this help?

Did your doctors or nurses help you find health information online?

- If yes, can you give me an example?
- Can you tell me why or why not the help that they gave you was actually helpful?

Part 4: Reflection and Future Engagement

If we haven't already talked about this, what are some ways learning about health online could be better for you?

How can your needs be met?

Do you have any further comments?
