# Supplementary material for: Digital Health Literacy in Pediatric Rheumatic Disease: A Qualitative Study Using Interpretive Description
Source: Children (Basel). 2026 Sep 18;13(9):1271. doi: 10.3390/children13091271 (PMC13605829; doi:10.3390/children13091271)
Supplement: Supplementary file 1 [file children-13-01271-s001.zip › File S4 - Caregiver Interview guide - DHL in PRD.pdf]

Interview Guide – Caregiver participants

Part 1: Background Information and Digital Health Engagement

1. Background Internet use

I'm interested in your regular online activities. Tell me about your day-to-day internet use.

How much time do you spend online each day?

- What sites or apps you are most comfortable using and why?
- How about social media – which ones do you normally use?

2. Current Use of Digital Health Resources and Motivation

What sort of information do or have you looked up online about your child's medical condition?

- Can you give me examples such as: information about the disease, treatments, side effects, or how to help your child at school?
- Tell me how you use social media to learn about your child's medical condition.

Can you tell me why you do or don't have a Saskatchewan MySaskHealthRecord account?

How do you find the information? For example, tell me how you search, what sites you use and how you choose which links to click?

If no: Probing questions:

- Please tell me what concerns and issues you have with searching for health information online?
- What would make you feel comfortable using the internet for your child's health?
- If no, what would need to change before you considered looking for health information online?

If sustained no:

- o Please tell me if you have issues understanding medical words and give me some examples?
- o Can you tell me if you struggle with understanding technology or computers and give me some examples?
- o Can you tell me what your concerns are with online privacy?
- o Can you tell me what your concerns are with online misinformation?
- o Can you tell me if you are worried that your child's health will get worse if you look for information online?
- o If you are comfortable, please tell me if your reluctance to go online is because you have issues affording internet, cell phone data plans, or devices to access the internet?

This is the end of the interview for those who don't use the Internet for health information and have no plans to start.

Part 2: Barriers to DHL

3. Perceived Challenges
Tell me about the issues you've had finding or using online health information about your child's health.

- Can you tell me about a time that you found some health information online that didn't help your child or made their health worse/not helped?
- Tell me how your experiences have changed the way you look for health information online.
- Have you ever felt overwhelmed, frustrated, or confused by the amount of health information available online? If so, can you tell me why you felt this way?
    - What did you do about this?
    - Have you ever looked for health information from another country or in another language? If so, tell me why, what country, and what language to looked for.
- Have any of your issues been because you struggle with technology? If so, can you tell me about it?
- Has paying for Internet access, cell phone plans, or devices has been an issue for you? If so, can you tell me about it?

4. Privacy
Do you have any privacy concerns with finding or sharing health information online?

- What steps do you take to deal with these concerns?

5. Misinformation
When looking for health information online, how concerned are you about misinformation?

- What does "misinformation" mean to you?
- What health information have you ignored because it may be misinformation?
- Has misinformation changed your confidence in caring for your child's medical condition?

Tell me how you decide if information is true and reliable?

Have your child's doctors or nurses disagreed with the information you found online? Or have the said something that disagrees with information you found online?

- Can you give me an example?
- What were the doctors'/nurses' concerns?

Part 3: Facilitators of DHL

6. Motivation for Digital Health Engagement: Now that we have talked about the negative, I'd like to discuss the positive aspects of looking for health information online.

What motivates you to look up medical information about your child on the Internet?

- What are hoping to learn?
- How do you hope this will help?

7. Perceived Benefits

Tell me about the good things you or your child have experienced from finding health information online?

- If yes, can you give me an example?
- Has the information you've found ever helped you make decisions about your child's health?

What advice would you give to other parents who are seeking health information online to help their child?

- Social media?

8. Usability

How can websites or apps better help parents find health information for their children?

9. Sharing of Information

How have you shared information your find online with others?

- What type of health information was it? Examples: dealing with side effects, improving their quality of life, parental advice.

Has anyone told you to stop finding or sharing any online information?

10. Improving Supports

If you were going to take a course or get training on the using the Internet for health, what would you want to learn?

Has anyone ever helped you find health information online?

- If yes, can you give me an example?
- What were the most and least useful things about this help?

Did your child's doctors or nurses help you find health information online?

- If yes, can you give me an example?
- Can you tell me why or why not the help that they gave you was actually helpful?

Part 4: Reflection and Future Engagement

What are some ways health information on the Internet could be made easier for you to find or understand?

How can your needs be met?

Do you have any further comments?
